# Supplementary material for: Unveiling the Bioactive Potential of the Invasive Jellyfish Phyllorhiza punctata Through Integrative Transcriptomic and Proteomic Analyses
Source: Biomolecules. 2025 Aug 4;15(8):1121. doi: 10.3390/biom15081121 (PMC12383608; doi:10.3390/biom15081121)
Supplement: Supplementary file 1 [file biomolecules-15-01121-s001.zip › Suplementary Material/FileS3/PEPFOLD4/XP_065055185_4306/clusters.html]

 PEP-FOLD clusters


Download archive of all models  here   

|  |  |  |
| --- | --- | --- |
| # | file | sOPEP |
| # cluster 1 - Size: 3 |  |  |
|  | XP\_065055185\_4306-model1 | -20.8628 |
|  | XP\_065055185\_4306-model1.1 | -20.4813 |
|  | XP\_065055185\_4306-model1.2 | -8.77265 |
| # cluster 2 - Size: 2 |  |  |
|  | XP\_065055185\_4306-model2 | -20.2585 |
|  | XP\_065055185\_4306-model2.1 | -19.8333 |
| # cluster 3 - Size: 1 |  |  |
|  | XP\_065055185\_4306-model3 | -17.1718 |
| # cluster 4 - Size: 4 |  |  |
|  | XP\_065055185\_4306-model4 | -16.2656 |
|  | XP\_065055185\_4306-model4.1 | -14.2536 |
|  | XP\_065055185\_4306-model4.2 | -11.0772 |
|  | XP\_065055185\_4306-model4.3 | -9.83367 |
| # cluster 5 - Size: 5 |  |  |
|  | XP\_065055185\_4306-model5 | -16.1097 |
|  | XP\_065055185\_4306-model5.1 | -16.0287 |
|  | XP\_065055185\_4306-model5.2 | -15.8224 |
|  | XP\_065055185\_4306-model5.3 | -15.7477 |
|  | XP\_065055185\_4306-model5.4 | -14.6037 |
| # cluster 6 - Size: 8 |  |  |
|  | XP\_065055185\_4306-model6 | -15.9706 |
|  | XP\_065055185\_4306-model6.1 | -15.8398 |
|  | XP\_065055185\_4306-model6.2 | -15.7572 |
|  | XP\_065055185\_4306-model6.3 | -15.7322 |
|  | XP\_065055185\_4306-model6.4 | -15.6877 |
|  | XP\_065055185\_4306-model6.5 | -15.5505 |
|  | XP\_065055185\_4306-model6.6 | -14.2263 |
|  | XP\_065055185\_4306-model6.7 | -13.3484 |
| # cluster 7 - Size: 4 |  |  |
|  | XP\_065055185\_4306-model7 | -15.7799 |
|  | XP\_065055185\_4306-model7.1 | -15.7764 |
|  | XP\_065055185\_4306-model7.2 | -15.4883 |
|  | XP\_065055185\_4306-model7.3 | -14.0594 |
| # cluster 8 - Size: 4 |  |  |
|  | XP\_065055185\_4306-model8 | -15.4193 |
|  | XP\_065055185\_4306-model8.1 | -15.0318 |
|  | XP\_065055185\_4306-model8.2 | -12.5317 |
|  | XP\_065055185\_4306-model8.3 | -8.42646 |
| # cluster 9 - Size: 1 |  |  |
|  | XP\_065055185\_4306-model9 | -13.7923 |
| # cluster 10 - Size: 3 |  |  |
|  | XP\_065055185\_4306-model10 | -13.6133 |
|  | XP\_065055185\_4306-model10.1 | -13.5645 |
|  | XP\_065055185\_4306-model10.2 | -10.6978 |
| # cluster 11 - Size: 5 |  |  |
|  | XP\_065055185\_4306-model11 | -12.6062 |
|  | XP\_065055185\_4306-model11.1 | -11.4508 |
|  | XP\_065055185\_4306-model11.2 | -9.50882 |
|  | XP\_065055185\_4306-model11.3 | -9.36277 |
|  | XP\_065055185\_4306-model11.4 | -9.00118 |
| # cluster 12 - Size: 1 |  |  |
|  | XP\_065055185\_4306-model12 | -12.6003 |
| # cluster 13 - Size: 1 |  |  |
|  | XP\_065055185\_4306-model13 | -12.3572 |
| # cluster 14 - Size: 2 |  |  |
|  | XP\_065055185\_4306-model14 | -12.1851 |
|  | XP\_065055185\_4306-model14.1 | -11.7036 |
| # cluster 15 - Size: 2 |  |  |
|  | XP\_065055185\_4306-model15 | -11.7469 |
|  | XP\_065055185\_4306-model15.1 | -10.949 |
| # cluster 16 - Size: 3 |  |  |
|  | XP\_065055185\_4306-model16 | -11.6304 |
|  | XP\_065055185\_4306-model16.1 | -10.3229 |
|  | XP\_065055185\_4306-model16.2 | -10.1044 |
| # cluster 17 - Size: 2 |  |  |
|  | XP\_065055185\_4306-model17 | -11.567 |
|  | XP\_065055185\_4306-model17.1 | -11.2383 |
| # cluster 18 - Size: 1 |  |  |
|  | XP\_065055185\_4306-model18 | -11.5117 |
| # cluster 19 - Size: 1 |  |  |
|  | XP\_065055185\_4306-model19 | -11.4716 |
| # cluster 20 - Size: 1 |  |  |
|  | XP\_065055185\_4306-model20 | -11.1387 |
| # cluster 21 - Size: 1 |  |  |
|  | XP\_065055185\_4306-model21 | -11.0918 |
| # cluster 22 - Size: 3 |  |  |
|  | XP\_065055185\_4306-model22 | -10.6546 |
|  | XP\_065055185\_4306-model22.1 | -10.2474 |
|  | XP\_065055185\_4306-model22.2 | -8.94524 |
| # cluster 23 - Size: 2 |  |  |
|  | XP\_065055185\_4306-model23 | -10.6412 |
|  | XP\_065055185\_4306-model23.1 | -9.43662 |
| # cluster 24 - Size: 1 |  |  |
|  | XP\_065055185\_4306-model24 | -10.6163 |
| # cluster 25 - Size: 1 |  |  |
|  | XP\_065055185\_4306-model25 | -10.364 |
| # cluster 26 - Size: 1 |  |  |
|  | XP\_065055185\_4306-model26 | -10.3294 |
| # cluster 27 - Size: 8 |  |  |
|  | XP\_065055185\_4306-model27 | -10.1993 |
|  | XP\_065055185\_4306-model27.1 | -10.1425 |
|  | XP\_065055185\_4306-model27.2 | -10.0873 |
|  | XP\_065055185\_4306-model27.3 | -9.46761 |
|  | XP\_065055185\_4306-model27.4 | -9.15863 |
|  | XP\_065055185\_4306-model27.5 | -8.82118 |
|  | XP\_065055185\_4306-model27.6 | -8.41032 |
|  | XP\_065055185\_4306-model27.7 | -7.93698 |
| # cluster 28 - Size: 10 |  |  |
|  | XP\_065055185\_4306-model28 | -10.1185 |
|  | XP\_065055185\_4306-model28.1 | -9.91327 |
|  | XP\_065055185\_4306-model28.2 | -9.34543 |
|  | XP\_065055185\_4306-model28.3 | -9.20182 |
|  | XP\_065055185\_4306-model28.4 | -9.14245 |
|  | XP\_065055185\_4306-model28.5 | -9.09249 |
|  | XP\_065055185\_4306-model28.6 | -8.5666 |
|  | XP\_065055185\_4306-model28.7 | -8.51873 |
|  | XP\_065055185\_4306-model28.8 | -8.33342 |
|  | XP\_065055185\_4306-model28.9 | -8.26901 |
| # cluster 29 - Size: 2 |  |  |
|  | XP\_065055185\_4306-model29 | -10.044 |
|  | XP\_065055185\_4306-model29.1 | -8.71496 |
| # cluster 30 - Size: 1 |  |  |
|  | XP\_065055185\_4306-model30 | -10.0203 |
| # cluster 31 - Size: 5 |  |  |
|  | XP\_065055185\_4306-model31 | -9.95325 |
|  | XP\_065055185\_4306-model31.1 | -9.32987 |
|  | XP\_065055185\_4306-model31.2 | -9.05937 |
|  | XP\_065055185\_4306-model31.3 | -8.49299 |
|  | XP\_065055185\_4306-model31.4 | -8.34953 |
| # cluster 32 - Size: 1 |  |  |
|  | XP\_065055185\_4306-model32 | -9.9105 |
| # cluster 33 - Size: 1 |  |  |
|  | XP\_065055185\_4306-model33 | -9.84913 |
| # cluster 34 - Size: 1 |  |  |
|  | XP\_065055185\_4306-model34 | -9.70902 |
| # cluster 35 - Size: 2 |  |  |
|  | XP\_065055185\_4306-model35 | -9.68117 |
|  | XP\_065055185\_4306-model35.1 | -9.33876 |
| # cluster 36 - Size: 3 |  |  |
|  | XP\_065055185\_4306-model36 | -9.25279 |
|  | XP\_065055185\_4306-model36.1 | -9.2311 |
|  | XP\_065055185\_4306-model36.2 | -8.74625 |
| # cluster 37 - Size: 1 |  |  |
|  | XP\_065055185\_4306-model37 | -9.20174 |
| # cluster 38 - Size: 1 |  |  |
|  | XP\_065055185\_4306-model38 | -9.0923 |
| # cluster 39 - Size: 1 |  |  |
|  | XP\_065055185\_4306-model39 | -9.02014 |
|  |  |  |
